# Photoperiod Regulates *vgf*-Derived Peptide Processing in Siberian Hamsters

**DOI:** 10.1371/journal.pone.0141193

**Published:** 2015-11-10

**Authors:** Barbara Noli, Carla Brancia, Roberta Pilleri, Filomena D’Amato, Irene Messana, Barbara Manconi, Francis J. P. Ebling, Gian-Luca Ferri, Cristina Cocco

**Affiliations:** 1 NEF-Laboratory, Dept. of Biomedical Sciences, University of Cagliari, Monserrato (CA), Italy; 2 Department of Life and Environmental Sciences, University of Cagliari, Monserrato (CA), Italy; 3 School of Life Sciences, University of Nottingham Medical School, Nottingham, United Kingdom; Kent State University, UNITED STATES

## Abstract

VGF mRNA is induced in specific hypothalamic areas of the Siberian hamster upon exposure to short photoperiods, which is associated with a seasonal decrease in appetite and weight loss. Processing of VGF generates multiple bioactive peptides, so the objective of this study was to determine the profile of the VGF-derived peptides in the brain, pituitary and plasma from Siberian hamsters, and to establish whether differential processing might occur in the short day lean state *versus* long day fat. Antisera against short sequences at the C- or N- termini of proVGF, as well as against NERP-1, TPGH and TLQP peptides, were used for analyses of tissues, and both immunohistochemistry and enzyme linked immunosorbent assay (ELISA) coupled with high-performance liquid (HPLC) or gel chromatography were carried out. VGF peptide immunoreactivity was found within cortex cholinergic perikarya, in multiple hypothalamic nuclei, including those containing vasopressin, and in pituitary gonadotrophs. ELISA revealed that exposure to short day photoperiod led to a down-regulation of VGF immunoreactivity in the cortex, and a less pronounced decrease in the hypothalamus and pituitary, while the plasma VGF levels were not affected by the photoperiod. HPLC and gel chromatography both confirmed the presence of multiple VGF-derived peptides in these tissues, while gel chromatography showed the presence of the VGF precursor in all tissues tested except for the cortex. These observations are consistent with the view that VGF-derived peptides have pleiotropic actions related to changing photoperiod, possibly by regulating cholinergic systems in the cortex, vasopressin hypothalamic pathways, and the reproductive axis.

## Introduction

Siberian hamsters are seasonal mammals that are hyperphagic in the summer and accumulate large fat reserves, but in response to short photoperiods they reduce food intake and body weight by catabolizing abdominal fat depots as a strategy to survive winter [1–2]. They provide a natural animal model to investigate how the mammalian brain is capable of generating a long-term suppression of appetite [3]. Analysis of hypothalamic gene expression in hamsters revealed photoperiodic regulation of *vgf* mRNA abundance within the SCN [4] and in the posteriuor arcuate nucleus [5–7], where some neurons expressed both VGF mRNA and 5-hydroxytryptamine receptors [8]. The neurotrophin responsive *vgf* gene encodes a polypeptide precursor protein (VGF) that is widely expressed in the brain [9] and is processed to give rise to a number of peptides of low molecular weight [10]. VGF and its derived peptides are not only abundantly expressed in the brain, but also in the pituitary, adrenal, gut, and pancreas [11–12]. Two families of VGF-derived peptides, namely TLQP and NERP, have been found to have biological activity in the regulation of food/water intake and energy homeostasis [13–17]. In rats, NERP-1 and -2 have been found involved in water balance through the control of vasopressin release [14,16], while NERP-2 facilitates feeding by acting in the lateral hypothalamus (LH), possibly by increasing orexin activity [15]. In hamsters, acute intracerebroventricular administration of TLQP-21 caused a sustained reduction in food intake and body weight and decreased abdominal fat depots in the long-day (LD) state [17]. In a recent study carried out in a Chinese hamster ovary cell line (CHO-K1), the C3a Receptor (C3AR1) has been identified as a target of the TLQP-21 peptide [18]. Despite these recent advances in understanding, little is known about the site(s) in the brain nor the molecular mechanism(s) by which VGF-derived peptides are involved in the response of hamsters to a winter photoperiod. Hence, we used immunohistochemistry (IHC) and enzyme-linked immunosorbent assay (ELISA) to profile the distribution of the VGF-derived peptides in the brain, pituitary and plasma of male Siberian hamsters, in the LD *versus* short day (SD) adapted state. In addition, we also used both high-performance liquid (HPLC) and gel chromatography to investigate the actual nature of VGF-derived peptides in the tissues studied. Among the VGF peptides, we decided to examine those previously found to be involved in mechanisms related to reproduction or metabolism, as these physiological processes display profound seasonal cycylicity in the hamster. TLQP family peptides have been examined in view of their specific role in the regulation of hamster food intake [17], PGH peptides for their cell-specific localization in the rat hypothalamic-pituitary-gonadal system [19], and NERP-1 because of its role in water balance through vasopressin regulation [14,16]. Furthermore, since the C- and N-terminus antibodies have been raised against the two extremes of the VGF precursor protein, they have also been used throughout, because of their possibility to recognise the VGF precursor.

## Material and Methods

### Antisera

Specific antisera (Table 1) were previously produced against short sequences at the C- or N- termini of the rat /mouse or human VGF, and of several known/predicted VGF-derived products: TLQP, NERP-1, and PGH. Since the hamster peptide sequences were not available, we tested both the rat and human VGF C-terminus and PGH antisera for the presence of immunoreactivity in hamster tissue. For both antisera, pilot experiments revealed more robust staining using the human antisera, so we used the human VGF C-terminus and TPGH antisera in all subsequent experiments. Immunogen peptides were conjugated with bovine thyroglobulin or keyhole limpet hemocyanin, *via* either an additional N-terminal D-tyrosine residue (VGF C-terminus, NERP-1 and PGH peptides), or an additional C-terminal cysteine (TLQP and N-terminal peptides). Specificity was addressed in parallel *via* IHC and ELISA (Table 2). The relevant immunostaining of each VGF antiserum was prevented by pre-absorption with the corresponding peptide (up to 100 nmol/ml).

**Table 1:** Antisera and antibodies used in the study.

Antigen	Species	References	Dilutions
h. VGF Ct	Rabbit	Cocco *et al*. 2007 [34]	1:250K / 1:3000
		Brancia *et al*. 2005 [33]	
TLQP	Sheep	this paper	1:60K / 1:1000
TPGH	Rabbit	Cocco *et al*. 2010 [23]	1:60K / 1:1000
NERP1	Rabbit	D’Amato *etal*.2012 [16]	1:60K / 1:1000
r.VGF Nt.	Guinea pig	Cocco *et al*. 2010 [23]	1:400K / 1:800
h. FSH	Mouse	Berger *et al*. 1990[35]	1:500
r. GH	Monkey	A.F Parlow	1:600
LH	Sheep	Biomol / Enzo	1:600
r. PRL	Mouse	Berger *et al*. 1990[35]	1:600
r.PRL	Mouse	Berger *et al*. 1990[35]	1:500
ACTH	Mouse	Berger *et al*. 1990[35]	1:300
Somatostatin	Rabbit	Abcam	1:600
Vasopressin	Mouse	Chemicon	1:200–400
ChAt	Goat	Millipore	1:800
Orexin	Goat	St Cruz	1:300
TH	Mouse	Sigma	1:800

Ct: C-terminus; Nt: N-terminus; FSH: follicle-stimulating hormone; GH: growth hormone; LH: luteinizing hormone; PRL: prolactin; ACTH: adrenocorticotropic hormone; ChAt: choline acetyltranferase; TH: tyrosine hydroxylase; h: human; r: rat.

**Table 2:** VGF assay characterization.

Assay	Peptide	IC_50_	CV1	CV2	R
**hVGF Ct**	hVGF_607–615_ (IEHVLLRRP)^1^	10	3–4	9–11	100
	rVGF_603–612_				0.8
	rVGF_609–617_				0,4
**TPGH**	hVGF_419–427_ (RSQEETPGH)^1^	0.2	3–7	6–7	100
	hVGF_420–427_				250
	hVGF_419–428_				1
	rVGF_422–430_				20
	rVGF_422–431._				<0.05
**NERP1**	VGF_301-309_(QQGLAQVEA-NH_2_)^1^	2	2–4	9–11	100
	VGF_285-309-NH2_				80
	VGF_301-309_ (QQGLAQVEA)				<0.0001
	VGF_301-309_((QGLAQVEA**G**)				<0.0001
**rVGF Nt**	hVGF_23–30_ (APPGRPEA)^1^	10	3–7	4–7	100
	rVGF_24–31_				<0.005
	rVGF_4–240_				<0.005
**TLQP**	rVGF_556-564_ (TLQPPASSR)^1^	5	2–3	7–13	100
	rVGF_555-564_				48
	rVGF_557-564_				20
	rVGF_556-566_ (TLQP-11)				10
	rVGF_556-576_ (TLQP-21)				250
	hVGF_554-577_ (TLQP-24)				3

IC_50_: half-maximal inhibitory concentration, in pmol/ml; CV1 and CV2: intra and inter assay variation (**%)**, respectively; h: human; r: rat; Ct.: C-terminus; Nt.: N-terminus. ^1^peptide used for plate coating and assay standard. All of the antisera used for the VGF tissue quantification showed 100% cross-reactivity with the corresponding peptides. R: cross reactivity (**%).**

### Animals

Adult male animals (n = 40) were obtained from a colony of Siberian hamsters (*Phodopus sungorus*) maintained at the University of Nottingham Biomedical Services Unit [20]. All studies were carried out in accordance with the UK Animals (Scientific Procedures) Act of 1986 (project licence: PPL 40/3604) and approved by the University of Nottingham Animal Welfare and Ethical Review Board. Hamsters were group housed and maintained at approximately 21°C and 40% humidity, and were allowed *ad libitum* access to water and standard laboratory chow comprising of 19% protein, 45% carbohydrate, 9% fat (Teklad 2019, Harlan, UK). Animals were housed from birth in LD conditions of 16 hours light: 8 hours dark with lights off at 11:00 GMT. Groups of hamsters that were aged 3–4 months were then transferred to SD of 8 hours light: 16 hours dark with lights off maintained at 11:00 GMT for a further 12–13 weeks. Control hamsters were maintained in LD. Body weight and molt scores [20] were recorded from hamsters in both LD and SD every 2–3 weeks, and testicular weight was measured at the time of tissue collection to confirm the photoperiodic response (see S1 Data for additional information).

### Tissue samples

For immunohistochemical studies hamsters exposed to SD (n = 7) and age-matched hamsters that remained on LD (n = 7) were perfused between 08:00 and 12:00 GMT with 4% paraformaldehyde (40 g/l, in 0.1 mol/l PO_4_ buffer) and the entire brains and pituitary glands were removed from the skulls. All the samples were rinsed in PBS containing 70 g/l sucrose and 0.1 g/l NaN_3_ and placed within blocks using cryoembedding media (65–75 g/l PVA, 40 ml/l PEG 56–98, 10 ml Tween 20, 0.5–1 g/l NaN_3_; [21]), snap-frozen in melting freon cooled with liquid nitrogen, and stored in the vapor phase of a liquid nitrogen tank. The sections were prepared using a cold-knife cryomicrotome (Microm HM-560, Walldof, Germany, [22]), collected on slides coated with poly-l-lysine (Sigma, Milan, Italy) and stored at -80°C. We used 26 animals for ELISA, studies were conducted in two batches, one of n = 14 for pilot experiments and then n = 12 (for each photoperiodic condition) for the main experiments. The entire cortices, hypothalami and pituitaries were homogenized in phosphate−buffered saline (PBS: 0.01 mol/l PO_4_, pH 7.2–7.4, 0.15 mol/l NaCl, ~10 ml/g tissue) containing a protease inhibitor cocktail (P8340, Sigma-Aldrich, Schnelldorf, Germany) by using an Ultra-Turrax Homogenizer (Ika-Werke, Staufen, Germany), heated in a boiling water bath (10–15 min), and centrifuged (3,000 × g, 10–15 min). Blood samples were drawn into tubes containing ethylenediaminetetraacetic acid disodium salt (EDTA; 1.78 mg/ml) ad centrifuged (3,000 × g, 10 min). The plasma supernatants were kept frozen until use (-20°C or lower). Extracted tissues have been used for ELISA first, while the remaining LD samples were pooled into single samples to be used for HPLC and/or gel chromatography.

### Molecular characterization by gel chromatography and HPLC

Since the proVGF is a large protein of 66 kDa, composed of 617/615 amino acid residues (rat/human, respectively), it was not detectable after HPLC separation. Hence, we used a gel chromatography appraoach that can isolate high MW forms to reveal the VGF precursor, and used HPLC to reveal other smaller VGF peptides. Since each technique has different advantages and limitations, we made a further effort to compare the two techniques, analysing the small VGF peptides by both gel chromatography and HPLC in order to evalidate the findings. Pituitary and plasma was only analysed by gel chromatography due to the restricted volume of the samples left.

For gel chromatography analyses, hypothalamus (1 mL), cortex (0.8 mL), pituitary (3 mL) and plasma (1.2 mL) extracts, pooled from LD hamsters were loaded onto a Sephadex G-50S column (Sigma; 2cm^2^ x 1m). This column was equilibrated with 50 mM ammonium bicarbonate and eluted with the same buffer. A molecular weight (MW) marker kit (MWGF70, Sigma) was used for the column calibration. The collected fractions (3 mL) were reduced in volume with a Vacufuge Concentrator (Eppendorf, Milan, Italy) and assessed by ELISA. The overall recovery of loaded immunoreactivity ranged between 80% and 100%.

For HPLC, tissue extracts pooled from LD hamsters were analysed using a Surveyor HPLC system (TermoFisher, San Jose, CA, USA) coupled by a T splitter to a diode-array detector and an LCQ Advantage mass spectrometer (TermoFisher) using a Vydac C18 column (150x2.1 mm, with 5um particle diameter, Hesperia, CA, USA) and the following solutions: eluent A 0.056% (v/v) aqueous trifluoroacetic acid (TFA) and eluent B 0.050% (v/v) TFA in acetonitrile/water 80/20 (v/v). A linear gradient from 0% to 80% of B (32 min) and from 80% to 100% B (1 min) was used at a flow rate of 0.20 ml/min, and the T splitter resulted in a flow-rate of 0.13 ml/min toward the diode array detector and 0.07 ml/min toward the mass spectrometer. The diode array detector was set at 214 and 276 nm. Before HPLC separation, hypothalamus and cortex extracts (4 and 5 mL respectively, pooled from 7 LD hamsters) were both fractionated by using 30kDa cutoff Amicon Ultra devices (Merck Millipore, Tullagreen Carrigtwohill Co. Cork, Ireland). Fractions containing <30kDa molecular species were dried in a Vacufuge Concentrator (Eppendorf, Milan, Italy), redissolved in 0.5 mL of 0.1% aqueous TFA, and 100 uL of these solutions were fractionated by HPLC. Fractions collected every two minutes from the outlet of the diode array detector, were dried with a Vacufuge Concetrator, redissolved in PBS and analysed by ELISA. Rat TLQP-21 (CPC, San Jose, CA, USA), rat TLQP-62 (Primm, Milan, Italy), NERP-1 (QQGLAQVEA, Bachem, San Carlos, USA), and TPGH-8 (CPC, San Jose, CA, USA) standards were analysed by HPLC using the same conditions, and the extracted ion current peaks revealed by searching in the chromatographic profile their specific multiple-charged ions.

### Elisa

This was carried out as previously reported [23], briefly multiwell plates coated with the specific synthetic VGF peptides were incubated with the VGF antisera in parallel with tissue samples and standards (the same synthetic peptides used for immunizations) followed by the relevant biotinylated secondary antibodies (Jackson, West Grove, PA, USA) and the streptavidin-peroxidase conjugate (30 min) (Biospa, Milan, Italy). Each VGF assay was characterized using various synthetic peptides (Table 2). Data were expressed as mean ± SEM throughout. Statistical analyses were carried out by one-way ANOVA, followed by t-test as appropriate (StatistiXL software, www.statistixl.com), and a P < 0.05 was considered statistically significant (see S2 Data, for additional information).

### Immunohistochemistry

Information about the antisera used are summarised in Table 1. Sections were incubated overnight with each VGF antiserum either alone or together with certain hormone/trophic factors, while the relevant species-specific secondary antibody/s (from donkey, conjugated with Cy3 or Cy2: Jackson Immunoresearch Laboratories, West Grove, PA) were used to reveal the primary antibody labelling. Slides were coverslipped with PBS-glycerol, observed and photographed using BX41 and BX51 fluorescence microscopes (Olympus, Milan, Italy) equipped with Fuji S2 and S3 Pro digital cameras (Fujifilm, Milan, Italy). Routine controls included substitution of each antibody, in turn, with PBS, and the use of pre-immune sera.

## Results

### Physiological responses to photoperiod

In all studies, hamsters exposed to short days showed a progressive decrease in body weight, for example hamsters used in the ELISA study showed a significant (P<0.01) decrease in body weight by week 7 of exposure to SD. At the point of tissue sampling after 13 weeks exposure to SD, body weight had declined by 20% relative to their initial weight (P<0.001). At this point testicular weight had also significantly declined (P<0.001), and in all hamsters the winter molt had been initiated (P<0.001). All these physiological responses to photoperiod are visible in S1 Data. No examples of individuals failing to respond to SD occurred in any of the cohorts of hamsters used.

### Molecular characterization

**Fig 1:**
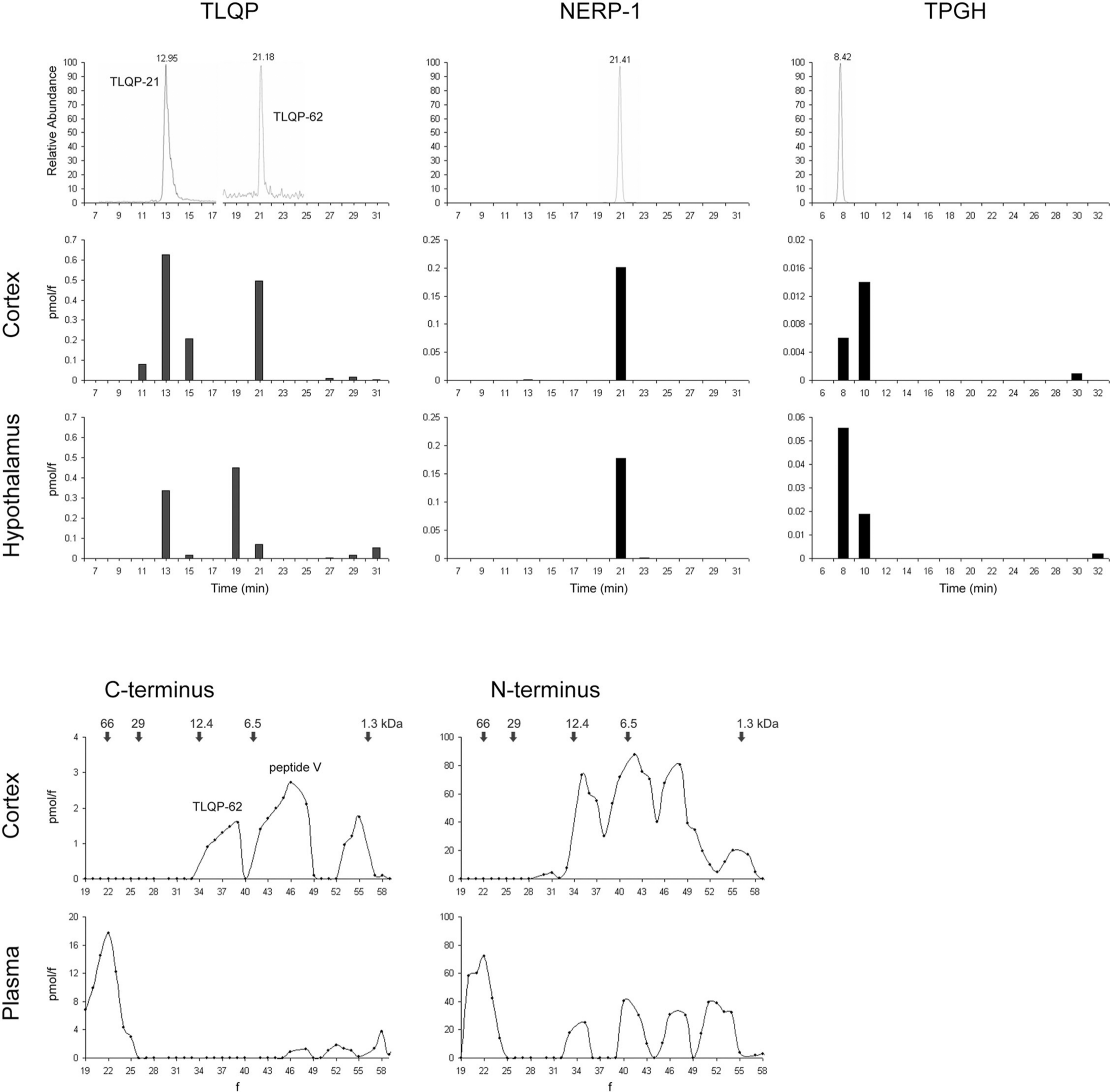
VGF molecular characterization. Upper panels: immunoreactivity deteced by ELISA after HPLC. Note that in the cortex, TLQP immunoreactivity was mainly found in fractions having the same elution times of synthetic TLQP-21 and TLQP-62. In the hypothalamus, TLQP-21 and TLQP-62 were also detected but a major further uncharacterized form eluted at 19 min. Immunoreactivity for NERP-1 was found exclusively in one fraction having the same elution time as synthetic NERP-1. TPGH immunoreactivty was found in one MW form eluting at the same time as the synthetic peptide but also in some uncharacterized fractions. Lower panels: gel chromatography revealed a large form of 60kDa that was recognised by both the antisera raised against the two extremes of the proVGF in plasma, but not in the cortex. In the cortex, two further MW forms were revealed by the C-terminus antiserum corresponding to TLQP-62 and peptide V.

In both cortex and hypothalamus samples, HPLC coupled with ELISA revealed that the immunoreactivity with TLQP antiserum was mainly found in fractions having the same elution times of the two synthetic standard peptides TLQP-21 and TLQP-62, with a major expression of the latter one in the cortex, compared to the hypothalamus (Fig 1, upper panel). In the same tissues, immunoreactivity for NERP-1 was found in the fraction having the same elution time of its corresponding synthetic peptide, while the TPGH antiserum labelled its equivalent peptide and a few uncharacterized fractions. Similar MW profiles for the above mentioned VGF peptides were also seen using gel chromatography coupled with ELISA, not only for the cortex and hypothalamus extracts, but also in pituitary extracts and plasma where nonetheless the TLQP-62 form was absent. Furtheromre, gel chromatography revealed a large form of 60kDa recognised by antisera raised against both the termini of proVGF. This was found in plasma and in the other tissues tested, with the only exception of the cortex (Fig 1, lower panel).

### VGF levels and changes in cortex, hypothalamus and pituitary

**Fig 2:**
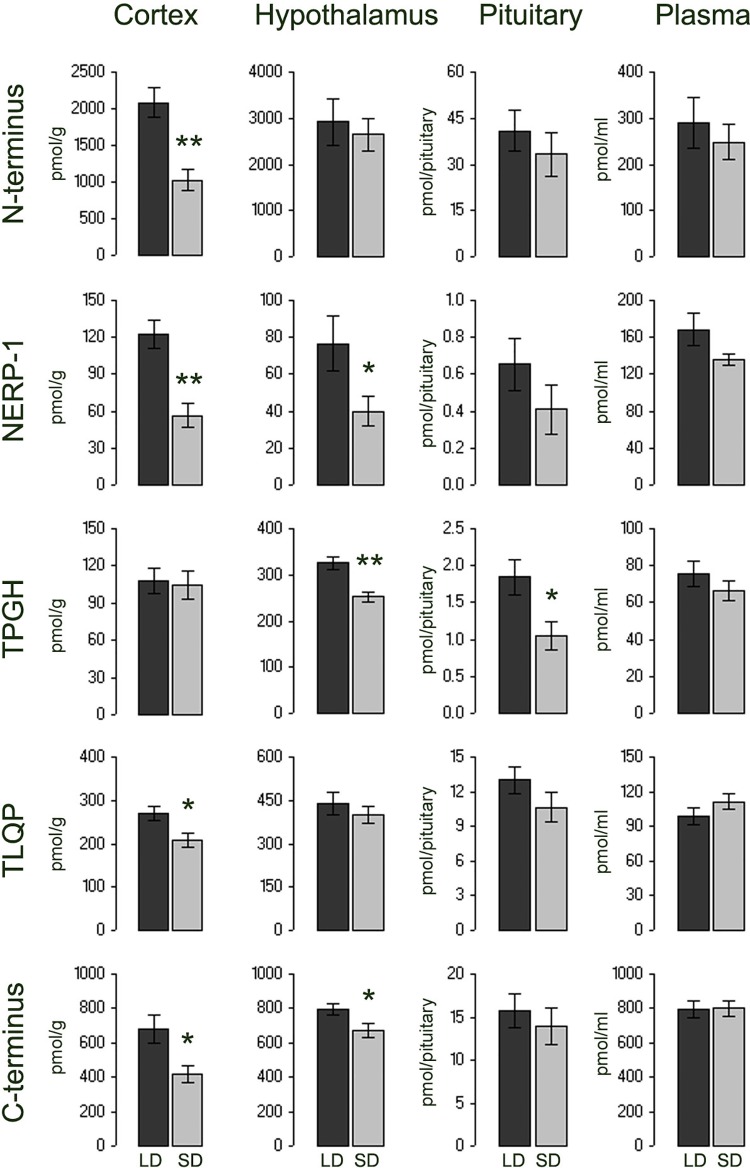
VGF peptides abundance as detected by ELISA. Values are group mean ± SEM. The short day (SD) produced a reduction in the VGF peptide levels that regards almost all the peptides in the cortex, NERP-1 and TPGH in the hypothalamus, and TPGH in the pituitary. [*p < 0.05, **p < 0.0005 vs long day (LD)]. N-terminus: p** = 0.0003; NERP-1:**p = 0.0003 and *p = 0.04; TPGH: **p = 0.0004, *p = 0.02; TLQP: *p = 0.008; C-terminus: *p = 0.01 in the cortex and 0.04 in the hypothalamus.

ELISA revealed that among the VGF peptides studied, concentrations of the VGF C- and N- terminal peptides were the most abundant in all tissues tested including plasma (Fig 2). In the hamsters kept in SD, lower concentrations were found in the cortex for all VGF peptides with the only exception of the TPGH, as compared to hamsters exposed to LD. This was also the case in the hypothalamus for NERP-1, VGF C-terminus and TPGH peptides. In the same SD hamsters, the TPGH peptides were the only ones to be decreased in the pituitary, while none of the VGF peptide species differed in plasma between the two photoperiodic states. See S2 Data for additional information.

### VGF peptide localization in brain

**Fig 3:**
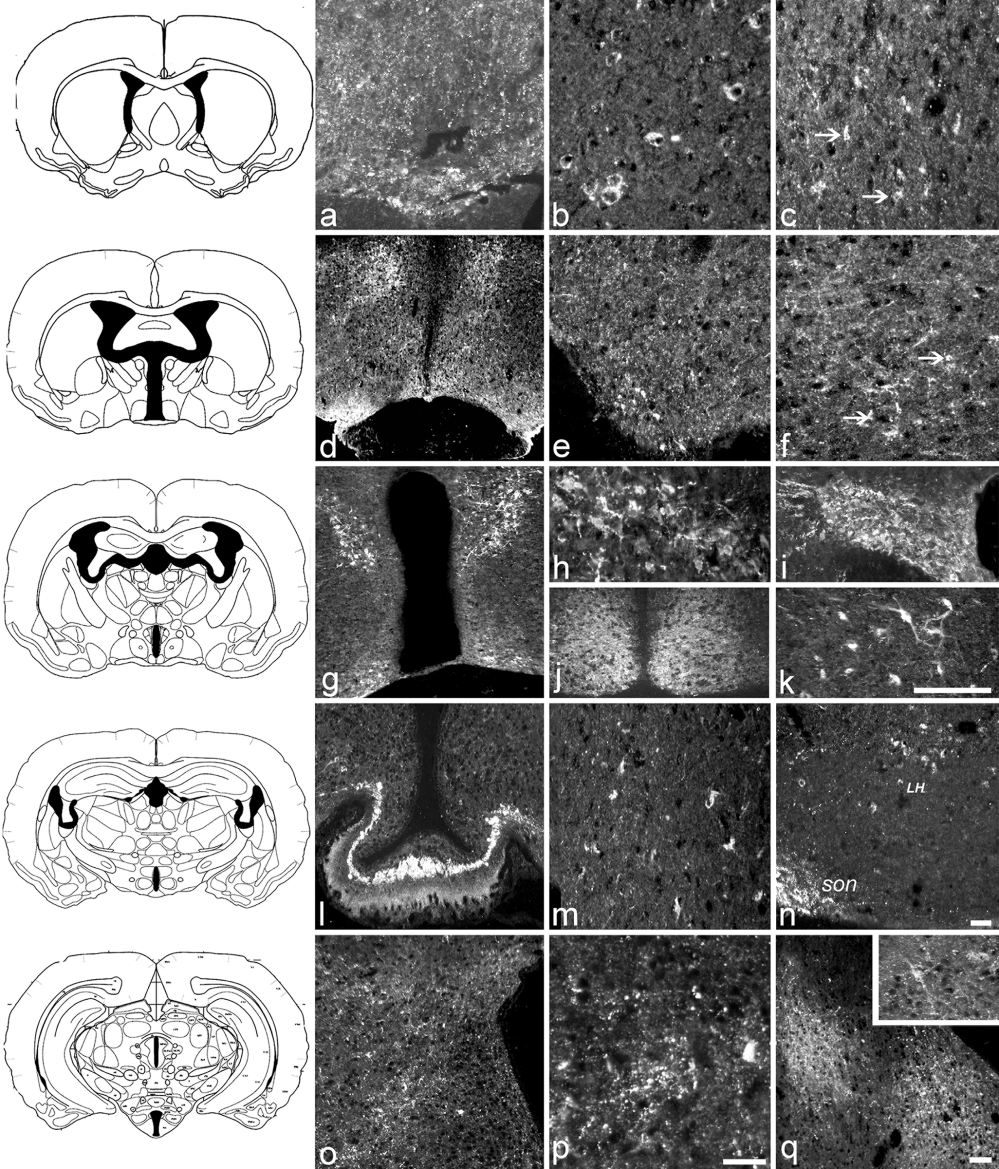
VGF C-/N- terminus peptide localization in the hamster brain. According to Morin and Wood, 2001 [36] coronal sections were stained at the level of (top to bottom) OVLT including area (a, b, c), preoptic area (d, e, f), supraoptic region (g, h, i, j, k), median eminence (ME, l, m, n) and arcuate nucleus (o, p, q, insert). VGF peptides are found in small axons and a number of nerve terminals of OVLT (a, C-terminus), and in the brain cortex within single punctuate cell bodies (b, N- terminus, frontal; c, C-terminus, parietal; arrows indicate small perikarya) as well as axons labelled by the VGF C-terminus only (c, C-terminus, parietal). Within the preoptic area, labelling was seen in the entire MPN (d, low magnification; C-terminus), within widely distributed axons and nerve terminals (e, f: C- and N- terminus, respectively), and small scattered perikarya (f, N- terminus arrows indicate the small perikarya). In the supraoptic region, labelling was abundant in many nuclei (g, low magnification, C- terminus) including Pa (h, C-terminus), SON (i, C- terminus) and SCN (j, N-terminus). In the same section, hippocampus CA3 (k, C- terminus) was rich in positive perikarya. ME (l, C- terminus) was labelled more intensively in the internal than in external layer, with Herring bodies brightly labelled. Perikarya were also visible in DM nucleus (m, C- terminus) and in an area just underneath the SON hence may compatible with LH (n, C- terminus). Immunopositive axons and nerve terminals (o, p; C- and N- terminus, respectively) were also found through the entire actuate nucleus and in amygdaloidei nuclei (q, C-terminus), in which scattered cell bodies were also visible (q, insert). Scale bars: a, c, f, m, p:100 μm; e, j, o, q: 100 μm; d, g, l, n: 200 μm; b, h, i, k, insert:100μm. SON: supraoptic nucleus, LH: lateral hypothalamic area.

We carried out the localization profile of the VGF peptides in the entire brain with a major focus on the cortex and hypothalamus, in view of the VGF modulations found by ELISA. The VGF C- and N- terminus antisera (Fig 3) produced almost identical patterns of immunoreactivity; both antisera generated the most robust immunostaining in terms of brightness, intensity and number of labelled cells, as compared to immunostaining for other VGF-derived peptides. The same antisera stained axons in the OVLT (Fig 3A), as well as cell bodies in different cerebral cortex areas including the frontal and parietal regions (Fig 3B and 3C identified by the arrows, respectively). Axons throughout the cerebral cortex were stained by the C-terminus antiserum, but they were sparsely distributed throughout the entire cortex area without an evident major concentration of fibers (Fig 3C). In the hypothalamus, the two VGF antisera revealed positive staining in the entire MPN (Fig 3D), within widely distributed axons and some small perikarya (Fig 3E and 3F), as well as in the MPO and the AvPe (not shown). A large number of perikarya were immunostained within the Pa (Fig 3G and 3H), SON, (Fig 3I) and SCN (Fig 3G and 3J), as well as in the hippocampus (Fig 3K).

In the ME (Fig 3L), the internal layer was more intensely stained than the external zone, comprising a dense population of immunoreactive terminals, with Herring bodies brightly labelled. A number of axons/nerve terminals and some scattered cell bodies were also stained in the AH, DM (Fig 3M), LH (Fig 3N), PH and VMH nuclei and through the entire arcuate nucleus (Fig 3O and 3P). Further areas were also labelled by both antisera, including the amygdaloid nuclei with a large number of axons and a few cell bodies (Fig 3Q), as well as the thalamus and substantia nigra (not shown).

**Fig 4:**
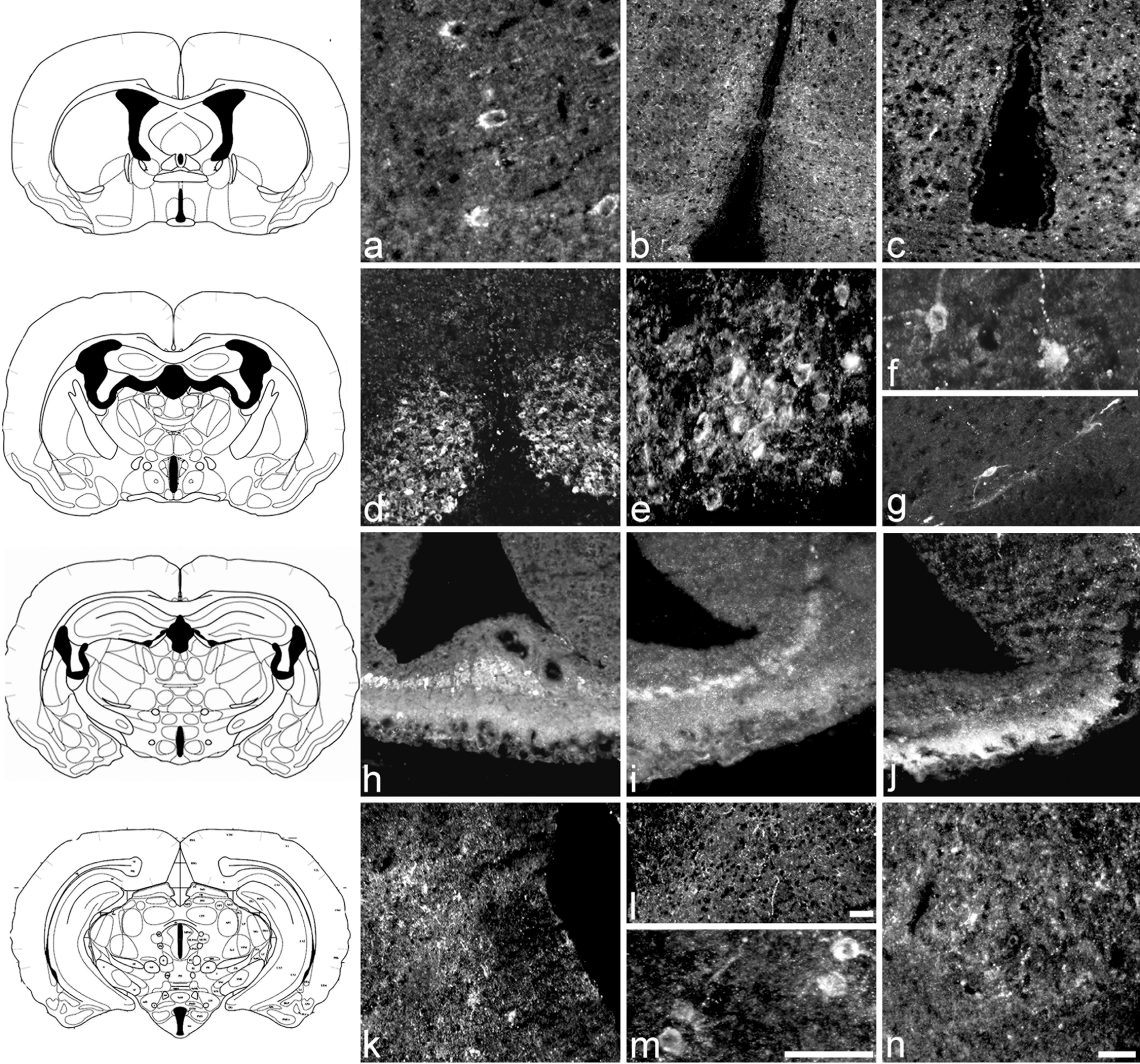
Localization of NERP-1, TPGH, and TLQP peptides in the hamster brain. Coronal sections are at the levels of (top to bottom) the preoptic area (a, b, c), supraoptic region (d, e, f, g), median eminence (ME, h, i, j) and arcuate nucleus (k, m, l, n). Cell bodies of the cortex were immunoreactive for NERP-1 peptides (a), this was also present in axons and nerve terminals of the MPN (b) while the TPGH peptides were present in small axons of MNP (c). In the supraoptic region, TLQP peptides were found in perikarya of the SCN (d, e: low and high magnitude, respectively), and NERP-1 peptides in a few cell bodies of the Pa (f) and SON (g). In the ME: the internal layer was more intensively stained than the external with NERP-1 (h) and TPGH (i) antisera, while TLQP peptides were more intensively labelled in the external layer (j). In the arcuate nucleus containing section, small perikarya, axons and nerve terminals were stained by the TPGH (k) and TLQP antisera (l: axons only), while TPGH antiserum labelled a few cell bodies and axons within the LH (m) as well as in amygdaloid nuclei (n). Scale bars: a, e, m: 100 μm; b, k, l: 100 μm; c, d, g, h, i, j, n: 100 μm.

When the other VGF antisera (NERP-1, TPGH and TLQP) were used (Fig 4), a less widespread staining pattern was seen compared to the high number of neuronal structures recognised by the two antisera raised against the extremities of the VGF precursor. However, a staining profile similar to that of the C-/N- terminus antisera was shown with the NERP-1, TPGH and TLQP antisera in the cortex, where strongly stained cell bodies were observed in most areas but not axons (Fig 4A). In the hypothalamus, NERP-1 antiserum labelled axons within AVpe, MPN area (Fig 4B), VMHM, and Mtu. TPGH immunoreactivity was found in a few cell soma in the AVpe, DM, PH, LH, and also in axons in the MPO (Fig 4C). Furthermore, these three VGF antisera labelled perikarya in the SCN (Fig 4D and 4E), a few cell bodies in the Pa and/or SON (Fig 4F and 4G), and, as expected, axons within the ME (Fig 4H–4J). In the latter area, VGF staining was much more intense in the internal than the external layer with NERP-1 and TPGH antisera (Fig 4H and 4I respectively) and *vice versa* with the TLQP antiserum (Fig 4J). Small perikarya and axons were stained by the TPGH (Fig 4K) and TLQP (Fig 4L: axons only) antisera in the arcuate nucleus, while TPGH labelled cell bodies were found within the LH (Fig 4M) and the PH as well as in several amygdaloid nuclei (Fig 4N). There was no clear evidence of differences in the VGF peptide distribution between the SD and LD state.

### Phenotype of VGF-immunoreactive neurons

**Fig 5:**
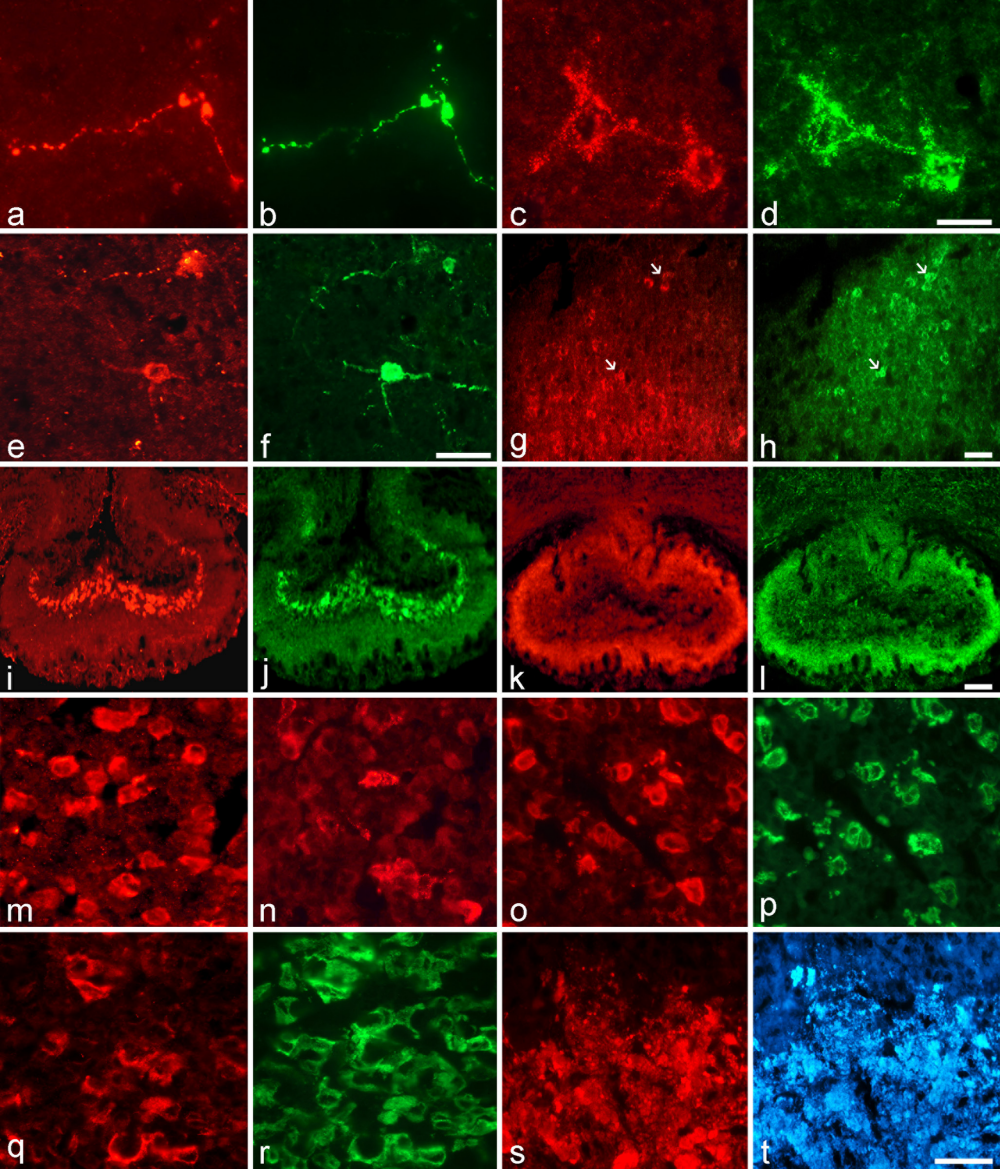
Colocalization profiles of the VGF peptides in hamster brain and pituitary. In the cortex, VGF C-terminus antiserum stained axons (a, red labelling) also positive for orexin antibody (b, green labelling), as well as perikarya (c, red labelling) containing ChAT (d, green labelling). In hypothalamic nyclei, NERP-1 (e, red labelling) and TLQP (g, red labelling) peptides, respectively in the Pa and SCN, were present in vasopressin cell bodies (f and h, respectively; green labelling, arrows identify colocalized cells). In the ME, NERP-1 (i, red labelling) and TLQP (k, red labelling) antisera stained vasopressin (j, green labelling) and somatostatin (l, green labelling) containing axons, respectively. In the anterior pituitary, VGF C-terminus antiserum labelled more cells in the LD than SD state (m *versus* n, respectively; red labelling). VGF C-terminus positive cells were found to contain LH (o versus p; red and green labelling, respectively) or ACTH (q *versus* r; red and green labelling, respectively). In the posterior pituitary, VGF C-terminus antiserum (s, red labelling) stained almost all vasopressin axons (t, blue labelling). Red, green and blue labelling reflects Cy3, Cy2 and AMCA, respectively. Scale bars: a, b, e, f: 100 μm; c, d: 50 μm; g–l: 100 μm m–t: 50 μm.

Dual staining experiments were carried out combining the various VGF antisera together with specific neurotransmitters and /or neuropeptides (Fig 5), with a major focus on the cortex and hypothalamus. In the cortex, exclusively VGF C-terminus antiserum (Fig 5A) labelled a few axons containing orexin (Fig 5B), while all VGF peptides studied were found within almost all perikarya corresponding to cholinergic interneurons (Fig 5C *versus* 5D, respectively). In the Pa, SON and SCN hypothalamic nuclei, the majority of vasopressin containing perikarya were immunostained with the C- and N- terminus antisera. A lesser proportion of cells was stained with the other VGF antisera, as it was the case of a few vasopressin containing cell bodies in the Pa (Fig 5E and 5F) and SCN (Fig 5G and 5H), stained by the NERP-1 and TLQP antisera, respectively. In the same nuclei, a very few orexin positive axons (not shown) were found to contain the C-/N- terminus and TPGH peptides, in the Pa, and VGF N- terminus peptides in the SCN (not shown). Consistent with the co-localization profile seen in the SON and Pa hypothalamic nuclei, the vasopressin axons of the internal ME were labelled by all VGF antisera. A most intense staining was found using the VGF C-terminus and NERP-1 antisera (Fig 5I and 5J), but less so with the TLQP antiserum that instead brightly labelled somatostatin containing axons within the external ME (Fig 5K and 5L). A very few orexin axons in the internal ME were also stained by TPGH antiserum (not shown).

### VGF peptide localization in the pituitary gland

All the VGF antisera studied stained the anterior pituitary with different degrees of intensity and numbers of labelled cells (Fig 5M–5T). The VGF C-terminus antiserum stained a reduced number of endocrine cells in SD compared to those stained in LD (Fig 5N *versus* 5M, respectively). Double label studies revealed that the majority of the endocrine cells stained by the various VGF antisera were gonadotrophs (Fig 5O *versus* 5P), with different degrees of co-localization, while 5–10% of cells stained by VGF C-/N- terminus, TPGH and NERP-1 antisera contained ACTH (Fig 5Q *versus* 5R). The posterior pituitary was stained by all VGF antisera with different intensities, but the C- (Fig 5S) and N- terminus as well as TLQP antisera showed the most intense labelling. As expected, all the VGF peptides co-localized with vasopressin in the posterior pituitary (Fig 5S *versus* 5T).

## Discussion

The localization and abundance of VGF-derived peptides have not so far been described in the Siberian hamster, as previous studies focused on either *vgf* gene expression, or the behavioural and physiological effects of TLQP-21 administration. This study provides evidence that VGF-derived peptides are widely distributed through the entire endocrine and nervous system, but in specific cell populations, some of which are down-regulated after SD exposure. Regulation of *vgf* mRNA by photoperiodic changes has been already reported previously in the SCN and in a small subsection of the arcuate nucleus (the dorsomedioposterior ARC) [4–7]. Our current analysis of peptide immunoreactivity indicates a regulation of VGF post-translational processing in the entire hypothalamus but also in further areas including the cortex, and pituitary, hence with more consistent finding compared to the other reported. Both HPLC and gel chromatography coupled with ELISA, revealed the specificity of our antisera in recognising MW forms compatible with the synthetic peptide standards. A few additional MW forms were also recognised by the TLQP and TPGH antisera, suggesting greater VGF post-translational processing resulting in uncharacterized forms containing the same sequences. In response to SD photoperiod, there was a down-regulation of VGF abundance in the cortex that was not so much evident in the other tissues tested, suggesting that VGF peptides could exert some roles in cortical mechanisms related to seasonal changes in behaviours. These data are strengthened by the absence of the VGF precursor in the cortex, suggesting an increase in processing mechanisms within this area compared to the hypothalamus and pituitary where proVGF immunoreactivity is still detectable. We also identified by both HPLC and gel chromatography that TLQP-21 and TLQP-62 are the major forms expressed in the cortex. TLQP-62 has been found to be involved in neural mechanisms occurring in the hippocampus, where it enhances neurogenesis [24], and synaptic activity [25–26]. We revealed TLQP peptides together with the other VGF peptides to be colocalized exclusively within cholinergic interneurons in the cortex. Cholinergic interneurons in the cortex have been found to play an important role in neurophysiological activities, such as learning, memory, arousal and sleep [27], so it is tempting to speculate that VGF-derived peptides may contribute to seasonal changes in these functions. The down-regulation of VGF processing in hamsters in the SD state was not only seen in the cortex but also in the hypothalamus with respect to NERP-1, C-terminus- and TPGH- peptides. All VGF peptides that changed in the hypothalamus are widely expressed in the hypothalamus-pituitary vasopressin system, suggesting a possible role of these peptides in seasonal changes in water retention through vasopressin regulation, a function previously identified in rats for the NERPs [14, 16]. During the SD state, previous studies in hamsters showed an up-regulation of the VGF mRNA in the dorsomedial posterior part of the arcuate nucleus, [5]. However, the same studies also showed a decrease in the remaining parts of the same nucleus [5], consistent with the immunoreactivity dow-regulation that we found elsewhere in the hypothalamus. It has had previously demonstrated [4], that light pulses can acutely upregulate vgf mRNA in the SCN of the Syrian hamster, so the effects of circadian rhythmicity and differential light exposure might contribute to the differences in VGF observed in the current study between hamsters in LD and SD, however hamsters from both photoperiods were euthanized towards the end of the light period to ameliorate such effects. We did not find any evidence for photoperiodic regulation of TLQP peptides, which is unexpected in view of their localization in the arcuate nucleus and in view of the role of TLQP-21 in food intake and body weight identified experimentally [17]. However, data regarding the precise hypothalamic structures in which TLQP-21 acts to influence food intake are not available, and if its action is limited to one specific nucleus, such changes would be difficult to detect using ELISA with extracts from the entire hypothalamus. Interestingly, the TLQP-21 together with TLQP-62 seems to be related to cortical mechanisms, since we found that TLQP peptides changed in the cortex, where they were found in cholinergic interneurons. This raises the possibility that its effects on food intake and body weight could be exerted through cortical acetylcholine related mechanisms [27]. Other than the vasopressin and the cholinergic system, VGF is also present within orexin axons in both the cortex (C-terminus peptides only) and internal ME (for both C-terminus and TPGH peptides), and even in the SCN (for N-terminus only). While orexin has been reported to be present in Syrian hamster into the above mentioned areas with a limited distribution [28], cell soma expressing orexin mRNA are restricted to the lateral hypothalamus area in the Siberian hamster [29]. There is evidence that orexin is involved in a wide variety of physiological and behavioral processes, including sleep, feeding, and endocrine regulation [30] so given the colocalizatin of VGF-derived peptides and orexin, the seasonal changes in TPGH and C-terminus peptides in the hypothalamus may be well related to these functions. In the pituitary gland, decreased immunostaining of TPGH peptides in SD was detected in gonadotrophs, suggesting an involvement of these peptides in the very marked photoperiod-induced changes in reproductive function in Siberian hamsters. Little information is available concerning the function of the TPGH family peptides, though their presence has been detected in neuroblastoma cells [31] and they have been found to decrease in the post-mortem human cortex from neurodegenerative disease patients [23]. In previous studies through female rats, TPGH peptides were largely found in the GnRH hypothalamus system and in the pituitary LH cells, but they were not modulated across the estrous cycle [19]. Hence, their decrease in pituitary from SD male hamsters may be related to the seasonal cessation of reproductive function.

## Conclusions

The changes in the VGF-derived peptide levels we observed after exposure to SD photoperiod likely reflect decreased activity of the VGF peptides in specific cells where they could modulate the release and/or production of other neurotransmitter/hormones that underlie seasonal cyclicity. Several lines of evidence support the view that VGF-derived peptides have cell-specific regulatory actions. For example, NERP peptides localized in the hypothalamic vasopressin cell bodies of male rats appear to be able to regulate vasopressin release [14], and their hypothalamic levels increase after water deprivation and salt load [16]. Similarly, in female rats, TLQP peptides localized in the FSH cells of the pituitary [19] can modulate FSH release [32], and their pituitary levels change during the oestrous cycle [19]. Our current results raise the possibility that changes in VGF peptide levels relate to regulation of cholinergic neuron activity in the cortex, and to vasopressin and reproductive hormone release in the hypothalamus and pituitary, respectively. Although we found VGF peptides to be abundant in the hamster circulation, their concentrations did not vary significantly with photoperiod, and their endocrine function remains to be elucidated.

## Supporting Information

S1 Data Hamster values. Data referr to body weight as well as testis weight and pelage score for one of the cohorts of hamsters used.
(PPTX)

S2 Data Elisa. Data referr to the values obtained by ELISA (pmol/g) of each animal for each VGF peptide analysed, in the cortex, hypothalamus, pituitary and plasma.
(JPG)

## Abbreviations (brain areas from the stereotaxic atlas)

AH: anterior hypothalamic area
AVpe: anteroventral periventricular nucleus
DM: dorsomedial hypothalamic nucleus
LH: lateral hypothalamic area
ME: median eminence
MPN: medial preoptic nucleus
MPO: medial preoptic area
Mtu: medial tuberal nucleus
OVLT: organum vasculosum of the lamina terminalis
Pa: paraventricular hypothalamic nucleus
PH: posterior hypothalamic area
PMV: premammillary nucleus
SCN: suprachiasmatic nucleus
SON: supraoptic nucleus
VMH: ventromedial hypothalamic nucleus
VMHM: ventromedial hypothalamic nucleus

## References

[1] Bartness TJ, Hamilton JM, Wade GN, Goldman BD (1989) Regional differences in fat pad responses to short days in Siberian hamsters. Am J Physiol 257:1533–40. 10.1152/ajpregu.1989.257.6.R1533 2604008

[2] Bartness TJ (1995) Short day-induced depletion of lipid stores is fat pad- and gender-specific in Siberian Hamster. Physiol Behav 58(3):539–50. 8587963 10.1016/0031-9384(95)00082-t

[3] Ebling FJ (2014) On the value of seasonal mammals for identifying mechanisms underlying the control of food intake and body weight. Horm Behav 66(1):56–65. 10.1016/j.yhbeh.2014.03.009 24681216 PMC4064697

[4] Wisor JP, Takahashi JS (1997) Regulation of the vgf gene in the golden hamster suprachiasmatic nucleus by light and by the circadian clock. J Comp Neurol 10;378(2):229–38. 9120062

[5] Barrett P, Ross AW, Balik A, Littlewood PA, Mercer JG, Moar KM, et al (2005) Photoperiodic regulation of histamine H3 receptor and VGF messenger ribonucleic acid in the arcuate nucleus of the Siberian hamster. Endocrinology 146 (4):1930–9. 15618354 10.1210/en.2004-1452

[6] Ross AW, Bell LM, Littlewood PA, Mercer JG, Barrett P, Morgan PJ, et al (2005) Temporal Changes in Gene Expression in the Arcuate Nucleus Precede Seasonal Responses in Adiposity and Reproduction. Endocrinology 146: 1940–1947. 15637286 10.1210/en.2004-1538

[7] Herwig A, Petri I, Barrett P (2012) Hypothalamic gene expression rapidly changes in response to photoperiod in juvenile Siberian hamsters (Phodopus sungorus) J Neuroendocrinol. 24(7):991–8. 10.1111/j.1365-2826.2012.02324.x 22487258

[8] Nilaweera KN, Archer ZA, Campbell G, Mayer CD, Balik A, Ross AW, et al (2009) Photoperiod regulates genes encoding melanocortin 3 and serotonin receptors and secretogranins in the dorsomedial posterior arcuate of the Siberian hamster. J Neuroendocrinol 21(2):123–31. 10.1111/j.1365-2826.2008.01810.x 19076271

[9] Van den Pol AN, Decavel C, Levi A, Paterson B (1989) Hypothalamic expression of a novel gene product, VGF: immunocytochemical analysis. J Neurosci 9(12):4122–37. 2556505 10.1523/JNEUROSCI.09-12-04122.1989 PMC6569627

[10] Trani E, Ciotti T, Rinaldi AM, Canu N, Ferri GL, Levi A, et al (1995) Tissue-specific processing of the neuroendocrine protein VGF. J Neurochem 65(6):2441–9. 7595538 10.1046/j.1471-4159.1995.65062441.x

[11] Ferri GL, Levi A, Possenti R (1992) A novel neuroendocrine gene product: selective VGF8a gene expression and immuno-localisation of the VGF protein in endocrine and neuronal populations. Mol Brain Res 13(1–2):139–43. 1315910 10.1016/0169-328x(92)90053-e

[12] Ferri GL, Noli B, Brancia C, D'Amato F, Cocco C. (2011) VGF: an inducible gene product, precursor of a diverse array of neuro-endocrine peptides and tissue-specific disease biomarkers. J Chem Neuroanatom 42(4):249–61. 10.1016/j.jchemneu.2011.05.007 21621608

[13] Bartolomucci A, La Corte G, Possenti R, Locatelli V, Rigamonti AE, Torsello A, et al (2006) TLQP-21, aVGF-derived peptide, increases Energy expenditure and prevents the early phase of diet-induced obesity. Proc Natl Acad Sci U S A 103(39):14584–9. 16983076 10.1073/pnas.0606102103 PMC1600003

[14] Yamaguchi H, Sasaki K, Satomi Y, Shimbara T, Kageyama H, Mondal MS et al (2007) Peptidomic identification and biological validation of neuroendocrine regulatory peptide-1 and -2. J Biol Chem 282(36):26354–60. 17609209 10.1074/jbc.M701665200

[15] Toshinai K, Yamaguchi H, Kageyama H, Matsuo T, Koshinaka K, Sasaki K, et al (2010) Neuroendocrine regulatory peptide-2 regulates feeding behavior via the orexin system in the hypothalamus. Am J Physiol Endocrinol Metab 299(3):394–401. 10.1152/ajpendo.00768.2009 20551287

[16] D'Amato F, Cocco C, Noli B, Cabras T, Messana I, Ferri GL (2012) VGF peptides upon osmotic stimuli: changes in neuroendocrine regulatory peptides 1 and 2 in the hypothalamic-pituitary-axis and plasma. J Chem Neuroanat 44(2):57–65. 10.1016/j.jchemneu.2012.05.001 22613228

[17] Jethwa PH, Warner A, Nilaweera KN, Brameld JM, Keyte JW, Carte WG, et al (2007) VGF-derived peptide, TLQP-21, regulates food intake and body weight in Siberian hamsters. Endocrinology 148(8):4044–55. 17463057 10.1210/en.2007-0038

[18] Hannedouche S, Beck V, Leighton-Davies J, Beibel M, Roma G, Oakeley EJ, et al (2013) Identification of the C3a receptor (C3AR1) as the target of the VGF-derived peptide TLQP-21 in rodent cells. J Biol Chem 288(38):27434–43. 10.1074/jbc.M113.497214 23940034 PMC3779738

[19] Noli B, Brancia C, D'Amato F, Ferri GL, Cocco C (2014) VGF changes during the estrous cycle: a novel endocrine role for TLQP peptides? PLoS One 9(10):e108456 10.1371/journal.pone.0108456 25280008 PMC4184793

[20] Ebling FJ (1994) Photoperiodic Differences during Development in the Dwarf Hamsters Phodopus sungorus and Phodopus campbelli. Endocrinology 95:475–482. 10.1006/gcen.1994.1147 7821784

[21] Cocco C, Melis GV, Ferri GL (2003) Embedding media for cryomicrotomy: an applicative reappraisal. Appl Immunohistochem Mol Morphol 11(3):274–80. 12966356 10.1097/00129039-200309000-00012

[22] Ferri GL, Cocco C, Melis GV, Aste L (2002) Equipment testing and tuning: the cold-knife cryomicrotome microm HM-560. Appl Immunohistochem Mol Morphol 10(4):381–6. 12607609 10.1097/00129039-200212000-00016

[23] Cocco C, D'Amato F, Noli B, Ledda A, Brancia C, Bongioanni P, et al (2010) Distribution of VGF peptides in the human cortex and their selective changes in Parkinson's and Alzheimer's diseases. J Anat 217(6):683–93. 10.1111/j.1469-7580.2010.01309.x 21039478 PMC3039181

[24] Thakker-Varia S, Krol JJ, Nettleton J, Bilimoria PM, Bangasser DA, Shors TJ, et al (2007) The neuropeptide VGF produces antidepressant-like behavioral effects and enhances proliferation in the hippocampus. J Neurosci 27(45):12156–67. 17989282 10.1523/JNEUROSCI.1898-07.2007 PMC3363962

[25] Alder J, Thakker-Varia S, Bangasser DA, Kuroiwa M, Plummer MR, Shors TJ, et al (2003) Brain-derived neurotrophic factor-induced gene expression reveals novel actions of VGF in hippocampal synaptic plasticity. J Neurosci 23(34):10800–8. 14645472 10.1523/JNEUROSCI.23-34-10800.2003 PMC3374594

[26] Bozdagi O, Rich E, Tronel S, Sadahiro M, Patterson K, Shapiro ML, et al (2008) The neurotrophin-inducible gene Vgf regulates hippocampal function and behavior through a brain-derived neurotrophic factor-dependent mechanism. J Neurosci 28(39):9857–69. 10.1523/JNEUROSCI.3145-08.2008 18815270 PMC2820295

[27] Oda Y (1999) Choline acetyltransferase: The structure, distribution and pathologic changes in the central nervous system. Pathol Int 49:921–937. 10594838 10.1046/j.1440-1827.1999.00977.x

[28] Mintz EM, Van den Pol AN, Casano AA, Albers HE (2001) Distribution of hypocretin-(orexin) immunoreactivity in the central nervous system of Syrian hamsters (Mesocricetus auratus). J Chem Neuroanat 225–238. 11382534 10.1016/s0891-0618(01)00111-9

[29] Reddy AB, Cronin AS, Ford H, Ebling FJP (1999) Seasonal regulation of food intake and body weight in the male Siberian hamster: studies of hypothalamic orexin (hypocretin), neuropeptide Y (NPY) and pro-opiomelanocortin (POMC). Eur J Neurosci 11(9):3255–3264. 10510189 10.1046/j.1460-9568.1999.00746.x

[30] Tsujino N, Sakurai T (2013) Role of orexin in modulating arousal, feeding, and motivation. Front Behav Neurosci 7:28 10.3389/fnbeh.2013.00028 23616752 PMC3629303

[31] Rozek W, Kwasnik M, Debski J, Zmudzinski JF (2013) Mass spectrometry identification of granins and other proteins secreted by neuroblastoma cells. Tumour Biol 34(3):1773–81. 10.1007/s13277-013-0716-0 23519838 PMC3661923

[32] Aguilar E, Pineda R, Gaytán F, Sánchez-Garrido MA, Romero M, Romero Ruiz A, et al (2013) Characterization of the reproductive effects of the VGF-derived peptide TLQP-21, in female rats: in vivo and in vitro studies. Neuroendocrinology 98(1): 38−50. 10.1159/000350323 23485923

[33] Brancia C, Nicolussi P, Cappai P, La Corte G, Possenti R, Ferri GL e (2005) Differential expression and seasonal modulation of VGF peptides in sheep pituitary. J Endocrinol 186:97–107. 16002540 10.1677/joe.1.05992

[34] Cocco C, Brancia C, Pirisi I, D'Amato F, Noli B, Possenti R, et al (2007) VGF metabolic-related gene: distribution of its derived peptides in mammalian pancreatic islets. J Histochem Cytochem 55(6):619–28 17312015 10.1369/jhc.6A7040.2007

[35] Berger P, Klieber R, Panmoung W, Madersbacher S, Wolf H, Wick G (1990) Monoclonal antibodies against the free subunits of human chorionic gonadotrophin. J Endocrinol 125(2):301–9. 1695661 10.1677/joe.0.1250301

[36] Morin LP, Wood RI, (2001). A Stereotaxic Atlas of the Golden Hamster Brain. Academic Press, San Diego.

