# Supplementary figures and images for: Photoperiod Regulates vgf-Derived Peptide Processing in Siberian Hamsters
Source: PLoS One. 2015 Nov 10;10(11):e0141193. doi: 10.1371/journal.pone.0141193 (PMC4640585; doi:10.1371/journal.pone.0141193)

## Slide 1
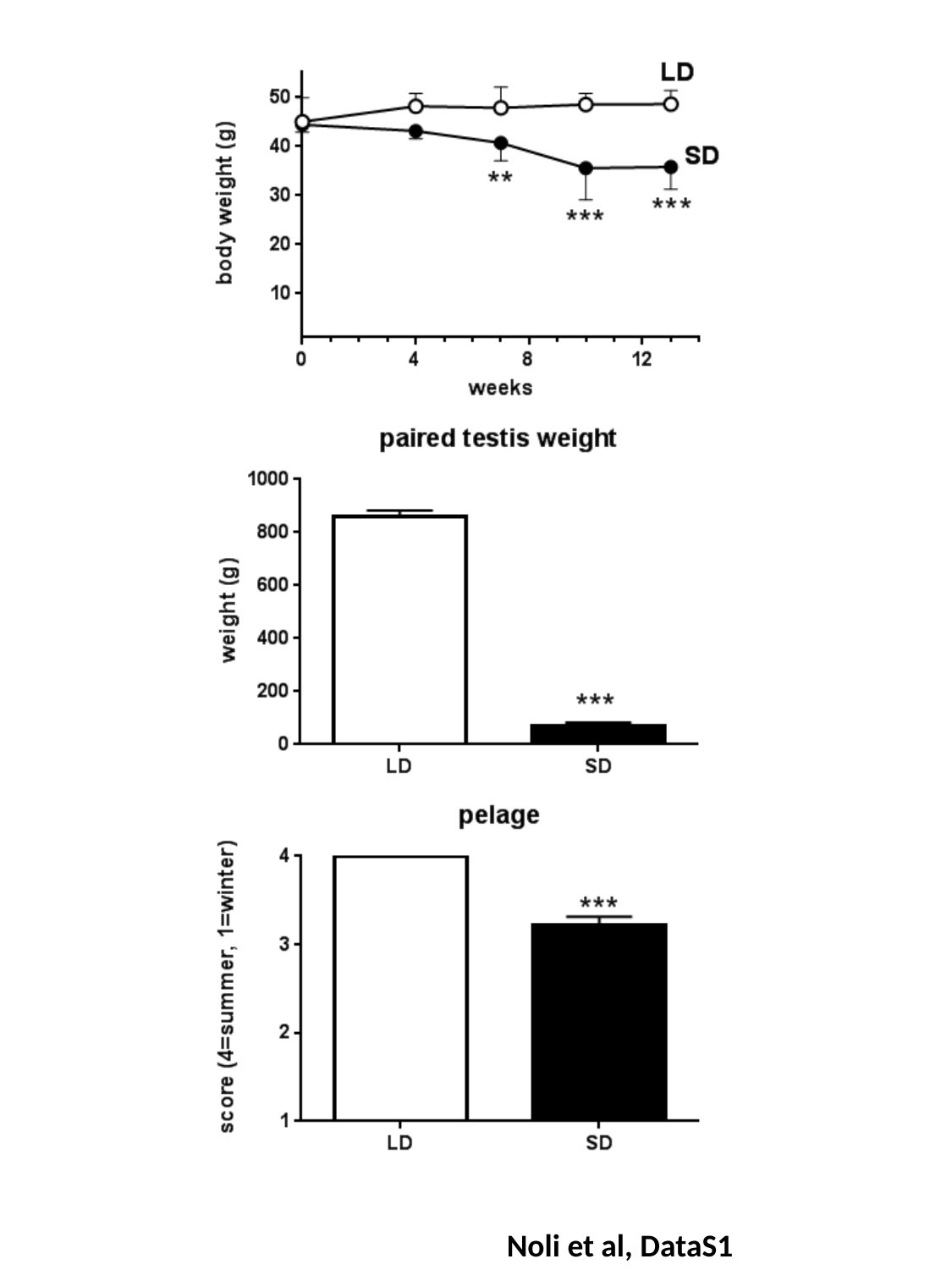

Noli et al, DataS1

Supplement: S1 Data — Data referr to body weight as well as testis weight and pelage score for one of the cohorts of hamsters used. (PPTX) [file pone.0141193.s001.pptx]

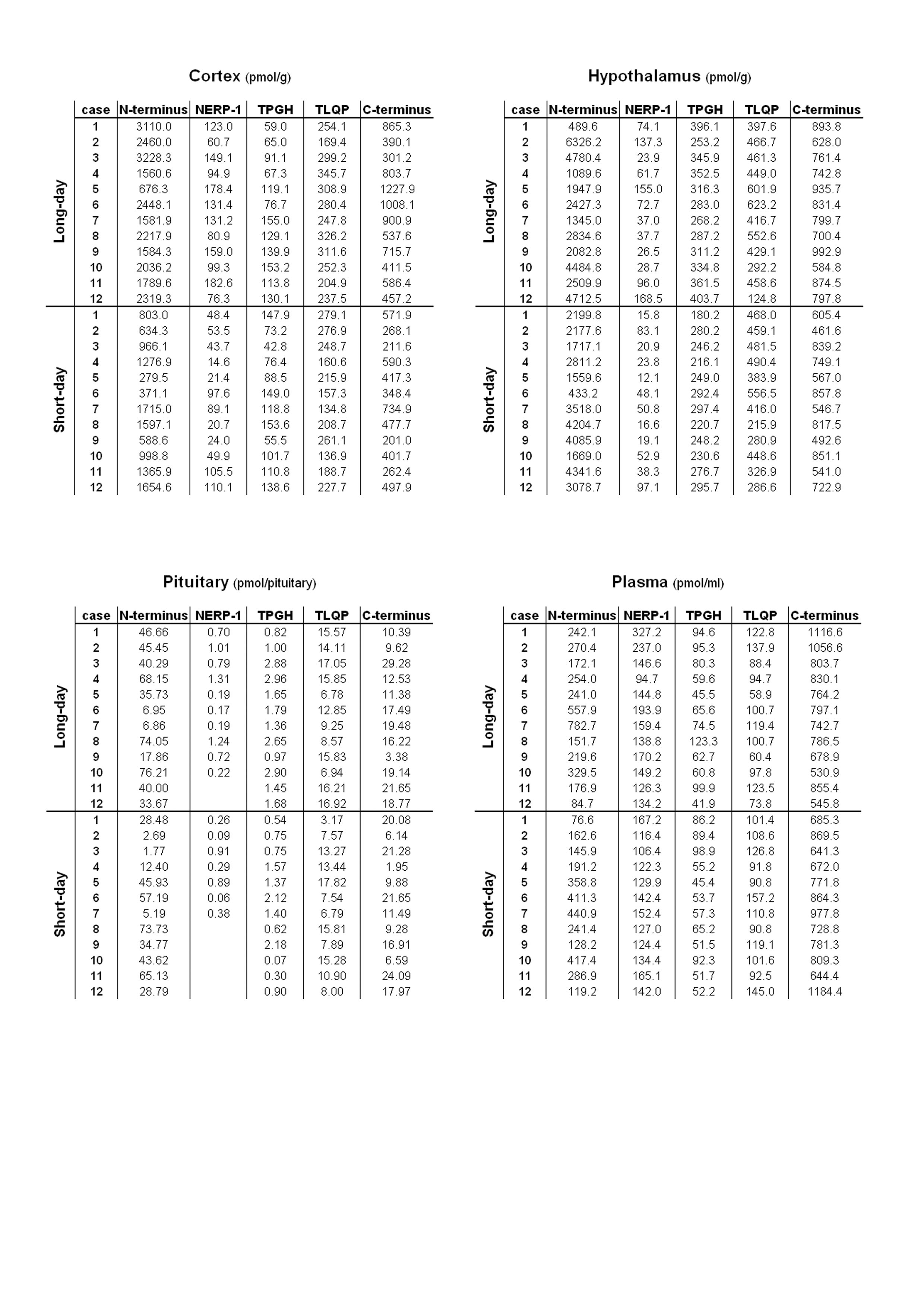

Supplement: S2 Data — Data referr to the values obtained by ELISA (pmol/g) of each animal for each VGF peptide analysed, in the cortex, hypothalamus, pituitary and plasma. (JPG) [file pone.0141193.s002.jpg]
